# Dr. J. Allen’s Review of Who Are Dentists

**Published:** 1861-08

**Authors:** 


### DR. J. ALLEN'S REVIEW OF WHO ARE DENTISTS.

In all branches of science or art facts should be chronicled and error discarded. With this view, we deem it proper to notice some of the errors in a series of articles recently published in the *Dental Register of the West*, and republished in other dental journals, under the head of "Who are Dentists?" by Dr. Wm. A. Pease. He tells us that

" Dentistry, as a profession, is of American origin. It had its rise in a great public want which nowhere but in the United States existed, and there is little likelihood that it ever will exist in any other country."

The idea here conveyed is, that teeth do not decay except in America. This is an egregious error. But he adds:

" The want that gave it birth gave it also a vigorous growth, unexampled in any other profession—for *dentistry* can not truly be said to date back much earlier than 1840. The Baltimore College had just been established, and it was thought that, by scientific investigation, means would soon be found to preserve the natural teeth, and thus greatly diminish, if not entirely dispense with the necessity of artificial substitutes."

Now if the Doctor will look at the history of dentistry, he will be able to trace it back more than 400 B. C. Hippocrates and Herodotus were among the Greek writers upon this subject; and from among the Romans we have also the writings of Pliny, Martial, Horace, Celsus, and others, which reflect much light upon this subject. And although as a science it was then in a rude state, yet Herodotus informs us that dentistry was practiced in Egypt as a distinct branch of surgery at that time; and corroborating evidences now exist in the fact that good gold fillings have been recently found in the teeth of mummies, which must have been inserted more than two thousand years ago.

Hippocrates informs us that the loss of the natural teeth was supplied with those of artificial production, made of bone or ivory, and also human teeth, secured in the mouth by means of ligatures made of flax, silk, gold, or silver wire, attached to other remaining teeth in the mouth. About one hundred and fifty years after Christ, Galen wrote a much better work on this subject than any of his predecessors, yet very little advancement had been made during the previous five hundred years. During the next fourteen hundred years, various authors wrote upon this subject, among whom were Ætius,

Phaxes, Albucasis, Eustachius, and others. In 1579, Pakie' a celebrated French surgeon, wrote a very correct treatise upon the teeth. He enjoyed a great reputation, and was ap- pointed surgeon in ordinary to Henry II., which office he held under three succeeding kings. French and English dentists had acquired celebrity in their native countries long before the Americans could claim supremacy in this branch of sur- gery, and we are much indebted to them for the light they have reflected upon our pathway in this department of sci- ence. From the writings of Eustachius, Parie, Hunter, Blake, Fox, Bell, Keocker, Leforgue, Duval, Leroy, and others, we have culled the basis of our profession; and Americans have done much, it is true, to elevate dental science, and a just tribute is now accorded to them by the Europeans, as well as those of our own country, for it. But to say that dentistry *originated* here in the United States, is a false assumption that should not be handed down to posterity in our dental records of the present era. No; the practice of dentistry was first introduced in the United States by Lemair, of the French forces, which joined our army during the revolution- ary war in 1778, after which he resumed his practice in this country. Mr. John Greenwood was the first American den- tist, and he established himself in the city of New York more than fifty years ago.

But Dr. Pease tells us, that soon after the establishment of the Baltimore College: "There followed an active course of experiments and investigations, to determine how far plugs would protect the teeth from further decay. Many of these experiments were necessarily imperfect and ill-directed; and it soon became obvious that people had more confidence in mechanical than *conservative* dentistry, and neglected their teeth. This neglect was ruinous. At last, in 1854, (it is well to be particular about dates), a rational theory was pro- mulgated for preventing ulceration and curing ulcerated teeth, and about the same time, as if to fix and make that the initial point for the *advent of dentistry as a profession*, several improvements were made in preparing gold for dental purposes, that enabled dentists to make much more dense and durable fillings, and also to build up and restore the form of broken teeth. *The dental profession was then fairly established.*" "In theory, at least, there was no longer any or but little need of *mechanics* or manufacturers of artificial sets of teeth."

Well might the dental profession then exclaim, "Eureka, Eureka!" The darkness which had so long obscured dentistry at last broke away, and the sun of dental science dawned upon this our happy land, as it nowhere else could shine. This great effulgence of light has revealed the principle by which a tooth can be plugged, and an ulcer cured. Dentistry is now fairly established, says the Doctor, and this is the sum total of what constitutes a dentist, according to his definition. He seems to have waked up, and fully appreciates the importance of these great principles, compared with which, the mechanic (as he calls him who inserts artificial dentures) sinks into insignificance, and his vocation is gone forever; for the Doctor says: "Now, after more than six years' practice of the new method of treating diseased teeth, dentists feel warranted in saying to their patients, there is no necessity for losing your teeth." Again he says: "Teeth can be so thoroughly plugged, that it will be as difficult to remove the plug as it would be to cut away a corresponding portion of the tooth. Even in those complicated cases where the tooth aches, where there is a swelling of the face, a gum-boil, or a discharge of pus, the pain can still be quieted and the ulcer healed."

No constitutional diathesis or difference in cases, is referred to as presenting any exceptions in this *new mode of practice*, but the reader is left to infer that it is infallible in all cases, and yet he tells us on page 282 Dental Register:— "Never warrant your operations for a day; make your plugs stick, but do not warrant them to do so."

If this important announcement had been made to the world one hundred years ago, and teeth could then have been preserved as effectually as the Doctor says they now can be, the following law against obtaining husbands under false pretenses, passed by the English Parliament in 1770, would have been unnecessary, so far as the teeth are concerned. This law reads thus :

"That all women, of whatever age, rank, profession, or degree,—whether virgins, maids, or widows, who shall after this act, impose upon, seduce, and betray into matrimony, any of his Majesty's male subjects, by virtue of scents, paints, cosmetic washes, artificial teeth, false hair, Spanish wool, iron stays or bolstered hips, shall incur the penalty of the law now in force against witchcraft, and like misdemeanors, and the marriage under such circumstances, upon conviction of the offending party, shall be null and void." But this is digressing.

Dr. Pease says there are two classes of dentists, or rather, there are dentists and mechanics, from whom very different treatment may be expected. The one, basing their practice on a knowledge of the human system and the laws that govern it, will preserve the natural teeth ; they will refuse to extract them merely because they ache, or there is a gum-boil at the roots. And he further adds, "The patients of the first preserve their teeth." Then why do they ache and have gum-boils ? The skillful physician and surgeon also base their practice upon their knowledge of the human system ; but they do not always preserve life and limb. No ; it is their knowledge of the human system and of the laws that govern it that forbids their making such unqualified assertions. It is the empirics who say they can always cure the sick, lame, and blind, under all circumstances. The patrons of the other class, he says, "get a shining set of white teeth, which every one knows to be artificial, in return for which they are but imperfectly cherished, the breath becomes offensive, the mouth falls in, the nose sticks further out, the lips shorter,—the lips and cheeks become wrinkled and shriveled, the cheek bones assume an unnatural prominence, and they look prematurely old. The one feeling little more responsibility resting upon them than what is common to mechanics, act accordingly —they persistently seek for a sale of their wares, talk loudly, and promise much; they obtrusively thrust forward their mechanical contrivances, and press pieces of artificial teeth on the attention of the public, all of which the other as studiously avoid. The one never rises above the customs of a craft or trade; the other is governed by the rules of a profession."

Although there may be some to whom the above remarks may be applied with truth, yet, to speak thus disparagingly of all who insert artificial dentures (and he makes no exceptions) evinces one of two things: either that the position he occupies in the profession is unfavorable to command a full view of it; or his zeal to eulogise one branch of dentistry at the expense of another, has warped his better judgment. If the Doctor will change his stand-point (say) to one a little more elevated, he will be able to see some dentists at least, in the distance, if not in his vicinity, who not only plug teeth and cure ulcers, but also insert artificial teeth, which, in point of appearance, are true to nature, and perform all the functions for which they were designed, as well as contributing largely to the comfort and gratification of their patients. The graphic description of artificial teeth, given by Dr. Pease, is evidently from his own practical knowledge of what they are, how they are made, and the final result.

When we review his three main points, first, that dentistry as a profession originated in this country, and that "it had its rise in a great public want, which nowhere but in the United States existed, and there is little likelihood that it ever will exist in any other country;" second, that the province or legitimate sphere of dentists is limited to plugging teeth and curing ulcers at the roots, and this established dentistry in 1854; and third, that those who insert artificial teeth are mere mechanics, nothing more, we are at a loss to define the stand-point from which he views the subject, and the more so because we have known the Doctor for several years, and believe him to be truthful, and writes what he conceives to be correct, and although we differ with him widely on the above points, still he has our respect and esteem.

(To be continued.)

